# Supplementary material for: Chromothripsis during telomere crisis is independent of NHEJ, and consistent with a replicative origin
Source: Genome Res. 2019 May;29(5):737–49. doi: 10.1101/gr.240705.118 (PMC6499312; doi:10.1101/gr.240705.118)
Supplement: Supplemental Material [file supp_gr.240705.118_Supplemental_file_1.zip › contigs/annotated_contigs/DB95/contig.5.DB95_length_1100_mean_cov_14.0363636364.docx]

**DB95_length_1100_mean_cov_14.0363636364**

```
ACAGAAATGGAAGCTAGAGAATCAAACCCCGAAAGATAA|TTTTACCTATTTGATGAATCAAGTATTCAGAAAAATGGACACTTACAAG
                                        >chr10:10589357-10589756 - E=7e-204
CTTATTGCTGAGATACACACACACACACACACACACACACTCTCTCTCTCTCTCTCTCTATCTTTTTGGTGTTGTAAGAACAAGACAGT

GACATTTAGCGGAGGTGGGAATGGGTAGTGGTGATGCTGCCACAGAGACCAAGTGGCTCACATGAGGGTAGGTGACACCTGGTGATCTT

GGAGGCAACTGGACTATGTAAGAATGATTAAATACACCTTAAGGCAACACTGAGATATAAGGCTTAGAAATTCTTTTTGTGCGCTTTGC

TTTTTTGTCCTGTGCTATCCGTTTGAGAGGCATTCCAACATTCATCACTTGTTCACTCTGTCACAGGC|AAA|CCCACCATGTGTATTT
                                                                     >chr17:61968870-61
CGACAATGCTCATGGAAGAAATTCAGAATTGGTCACTTTAGGTTAGGGTAAATTCAAAAAAAATGACAGAGTCAGTAAAACATTATGCC
969118 + E=4e-137
AGGGGTCAAGGATGACTGGAGCTCTGCCTGCTGAACTCTTCCCTGTGCGAGTCTCACTAATCTTATCTTCAGGAAGAGTTTTCGATAGA

AAATACCATTAAGGGCCAGGCCTGGTGGCTCCTGCCTATGATCCTAT|CACT|ATCATCTTCGAAGAGGAGACCCTACTTCCAAGTCAG
                                                >chr22:49925622-49925665 - E=7e-14
GCC|TGCGGATGCGGAACATGGCCTGAGAGCAGTAACTCAGGTACAGGTCTTCATCCTCGGGTGTCAGCGTCACCTGGTTCTCCTGGTA
     >chr16:57764730-57764960 - E=2e-126
CCACTGCTGCTTCTTTTGCAGCTCCTGGGTCTCCTGTGGTCATAGGGCAGACAGAGAAGGAGGACTGACAAGGCTGACCTTCACCCAGG

CTGCAGGCTGCAATCCCATGGGTCACTAGACAGCACCATGACGGTTGGCCCCAG|TG|ACAGGTGTAGAACCAATTAAGGCCATTACTG
                                                       >chr10:10589156-10589318 - E=2e-
TGTCATGTGGGAATTAGGCATTCGGCACTGTGGGTCAGGTGGCTGGCTAGTGGGGGCTGCAGCTTGGGCACAATAGTGAGGAATTTGTC
85
ATGGATATTTCTCGATAGGCAAGTATTTGGGTAGGGCCAG
```
